# A combined experimental and theoretical study of radon solubility in fat and water

**DOI:** 10.1038/s41598-019-47236-y

**Published:** 2019-07-24

**Authors:** Elvira P. Sanjon, Andreas Maier, Annika Hinrichs, Gerhard Kraft, Barbara Drossel, Claudia Fournier

**Affiliations:** 1 Institut für Festkörperphysik, Technische Universität Darmstadt, Hochschulstr. 6, 64289 Darmstadt, Germany; 2 GSI Helmholtzzentrum für Schwerionenforschung, Biophysics division, Planckstr. 1, 64291 Darmstadt, Germany; 3 Goethe Universität Frankfurt, Max-von-Laue-Str. 1, 60438 Frankfurt, Germany

**Keywords:** Computational biophysics, Biophysical chemistry, Biological physics

## Abstract

Radon is a radioactive noble gas that can enter the human body, thus increasing the risk of lung cancer. But it is also used for treatment of various ailments, most notably rheumatoid arthritis. The accumulation of radon differs between tissues, with particularly high concentrations in fat tissue. To understand the underlying mechanisms, a combination of *γ*-spectroscopy and molecular dynamics simulations were performed, to study the accumulation of radon gas in contact with several liquids (water, fatty acids). The solubilities, specific for a defined radon activity concentration, are in good agreement and differ by two orders of magnitude between water and fat, caused by radon disrupting the hydrogen bond network of water. In contrast, the energy cost of introducing radon atoms into fat is low due to the dispersive interaction between radon and fat, which is a non-polar solvent. This correlation was also explicitly demonstrated in our simulations by changing the polarization of the solvent.

## Introduction

Radon is a naturally occuring radioactive noble gas. Its most stable isotope ^222^Rn has a half-life of 3.8 days. Exposure to radon and its decay products accounts for the largest proportion of annual radiation dose from natural sources^1,2^. The highest dose is deposited in the lung and is caused mainly by the short-lived radon daughter nuclei ^218^Po and ^214^Po^3,4^ which are *α*-emitters, thus increasing the risk of lung cancer^5,6^. Radon is inhomogeneously distributed in different tissues^7,8^, resulting in significant doses to other organs^3^. Despite this risk, radon has beneficial effects when used for therapy of inflammatory diseases such as rheumatoid arthritis and ankylosing spondylitis, but the underlying molecular mechanisms are not fully understood^9,10^.

Radon enters the body via the epithelial tissues of lung, skin and the gastrointestinal tract, diffuses into the bloodstream and accumulates in fatty tissue^7,11,12^. The physical and chemical reasons behind this inhomogeneous distribution in the human body have not been explored yet, although this is of great interest for understanding the effect of radon therapy as well as the potential health risks. In order to fill this gap, we propose that radon diffuses slower into liquid water due to the strong polar interaction between the water molecules. Fat in contrast is non-polar, which leads to an energetic lower threshold for radon to enter in fatty tissue. To check this hypothesis, we used a combination of *γ*-spectroscopy and molecular dynamics (MD) simulation to investigate the concentration specific solubility of radon in water and fat.

The sample/air partition coefficient of radon or concentration specific solubility (*S*) is defined by the relation

$$S=C/{{\rm{D}}}_{{\rm{Rn}}},$$

where *C* is the concentration of radon in the liquid and D_Rn_ is the radon density in the surrounding gas, which has been chosen in the simulations as a pure radon gas at room temperature and normal pressure. Previous simulations^13–18^ studied the diffusion of a small number (2–5) of noble gas atoms (He, Ne, Ar, Kr, Xe) in water in order to investigate the hydrophobic effect. They found that the bigger the atom, the more distorted is the local structure of the surrounding water molecules. Since the radon atom is even larger than these atoms used in previous studies, its presence in water must considerably disturb the local structure. There exist no comparable studies for the diffusion of a noble gas in a non-polar solvent.

We proceeded as follows: In the experiments, we determined the concentration specific solubility of radon by measuring the activity of its daughter nuclei via *γ*-spectroscopy and calculating back to the initial activity of radon, and by additionally measuring the radon concentration in air. In the MD simulations, we evaluated the concentration specific solubility of radon directly from its equilibrium concentration in the different types of liquids, and we explored additionally the influence of molecular polarity by artificially modifying the partial charges on the water molecule in order to test the idea that the polarization of the solvent is a main determinant of concentration specific solubility of radon.

## Results and Discussion

In experiments, we used an isotonic water-salt solution as a model for standard cells and two types of fats as a model for fat cells. A saturated and an unsaturated fatty acid were chosen in order to check whether the concentration specific solubility of radon depends on the type of fat characterized by different degrees of polarity.

In the simulations, we had to use fat molecules with shorter chains in order to achieve equilibration. Two types of fat and water-salt solutions were used. In addition, we also used hexane as well as pure water (labelled as Q_0_) and water with a polarization that was artificially reduced (Q_−_) or enhanced (*Q*_+_) by 15%.

Table 1 shows the density and nearest-neighbour distances for the different liquids used in the computer simulations. The attraction between water molecules is dominated by the electrostatic interaction, and therefore density decreases with decreasing polarity. The densities obtained for water Q_0_ (0.995 g · cm^−3^) and for water Q_+_ (1.058 g · cm^−3^) are close to the empirical value of ≈1.000 g · cm^−3^.

**Table 1:** Computer simulation results for the nearest-neighbour distance (R) of solvent molecules and solvent density (D).

	R (nm)	D (g · cm^−3^)
Water Q_+_	0.298	1.058
Water Q_0_	0.310	0.995
Water Q_−_	0.352	0.852
C_6_H_14_	0.446	0.662
C_4_H_8_O_2_	0.451	1.014
C_10_H_20_O_2_	0.463	0.921
Rn	0.453	9.36 · 10^−3^

The simulations have been performed at normal pressure p = 1 bar and room temperature T = 298 K. Data for the radon gas are added for comparison.

The concentration of radon in the different liquids is shown in Table 2. Radon is hardly soluble in water Q_+_ and water Q_0_, but accumulates to some extent in water Q_−_. More radon accumulates in alkaline solutions than in pure water, indicating that the effect of adding salt is similar to the effect of changing the polarity of water.

**Table 2:** Simulation results for the equilibrium radon concentration *C* in the different liquids, which are surrounded by radon gas with the properties given in Table 1 (with standard error).

Liquid	*C* (*g* · *cm*^−3^)
Water Q_+_	≈0
Water Q_0_	(6.451 ± 0.007) · 10^−4^
Water Q_−_	(2.093 ± 0.023) · 10^−3^
Water +5% NaCl	(1.372 ± 0.019) · 10^−3^
Water +1.9% CaCl_2_	(1.464 ± 0.016) · 10^−3^
C_6_H_14_	(1.333 ± 0.058) · 10^−2^
C_4_H_8_O_2_	(2.161 ± 0.090) · 10^−2^
C_10_H_20_O_2_	(2.260 ± 0.93) · 10^−2^

The experimental results obtained with oelic and linoleic acid and isotonic salt solution are shown in Table 3. The experiments for the salt solution and oleic acid were repeated three times, and four times for linoleic acid. The final results for concentration specific solubility of radon obtained from experiment and simulation are summarized in Table 4. They agree very well with each other for all the fatty acids, with concentration specific solubility values around 2. These values are of the same order of magnitude as measured by Nussbaum and Hursh^12^, and no influence of the chain length of the fatty acids could be determined.

**Table 3:** Experimentally determined radon concentration in the sample for the different nuclide ^214^Pb (C_*Pb*_) and ^214^Bi (C_*Bi*_) and radon activity-concentration D_Rn_ during experiments.

	C_*Pb*_	C_*Bi*_	D_Rn_
	(Bq/cm^3^)	(Bq/cm^3^)	(Bq/cm^3^)
H_2_O +0.9% NaCl	0.11 ± 0.08	0.09 ± 0.07	3.97 ± 0.11
	0.17 ± 0.01	0.17 ± 0.01	6.39 ± 0.21
	0.13 ± 0.01	0.14 ± 0.01	6.20 ± 0.20
C_18_H_34_O_2_	3.65 ± 0.08	3.62 ± 0.08	2.12 ± 0.07
	4.05 ± 0.08	3.96 ± 0.08	2.88 ± 0.08
	5.32 ± 0.08	5.26 ± 0.08	3.76 ± 0.10
C_18_H_32_O_2_	7.76 ± 0.22	7.36 ± 0.22	3.91 ± 0.11
	8.86 ± 0.22	8.36 ± 0.22	2.88 ± 0.08
	6.73 ± 0.21	7.07 ± 0.21	3.76 ± 0.10
	7.15 ± 0.21	6.90 ± 0.21	3.76 ± 0.10

The assumed densities are 1.000 g · cm^−3^ for the isotone solution, 0.895 g · cm^−3^ for oleic acid and 0.900 g · cm^−3^ for linoleic acid. The experiments were done at an air pressure of 1001 ± 9 mbar and a temperature of 295.2 ± 0.4 K (with standard deviation).

**Table 4:** (a) Measured concentration specific solubility of radon in oleic acid (C_18_H_34_O_2_), linolic acid (C_18_H_32_O_2_), and an isotone solution (with standard deviation).

Liquid	Concentration specific solubility of radon
**(a) Experiment**	
Water +0.9% NaCl	0.025 ± 0.003
C_18_H_34_O_2_	1.50 ± 0.16
C_18_H_32_O_2_	2.16 ± 0.52
**(b) Simulation**	
Water Q_+_	≈0
Water Q_0_	0.07 ± 0.02
Water Q_−_	0.22 ± 0.06
Water +5% NaCl	0.14 ± 0.05
Water +1.9% CaCl	0.15 ± 0.04
C_6_H_14_	1.42 ± 0.15
C_4_H_8_O_2_	2.3 ± 0.23
C_10_H_20_O_2_	2.4 ± 0.35

(b) Computer simulation results for concentration specific solubility of radon in pure water, pure water with increased (Q_+_) or reduced (Q_−_) polarization, two types of water-salt solutions, hexane (C_6_H_14_), butyric acid (C_4_H_8_O_2_), and capric acid C_10_H_20_O_2_ (with standard error). [For more details, see the methods section].

In water, concentration specific solubility is lower by a factor of approximately 100 in the experiments, and by a factor of the order of 20 in the simulations. For the Q_+_ water, the concentration of solved radon is so low that it could not be distinguished from 0 in the simulations. Because of this sensitive dependence of concentration specific solubility of radon on water polarization, we think that the difference between simulation and experiment is due to the fact that water molecules used in simulations have fixed partial charges that cannot respond to different types of interaction partners. In any case, the computer simulations confirm that concentration specific solubility of radon differs vastly between polar and non-polar liquids.

**Figure 1:**
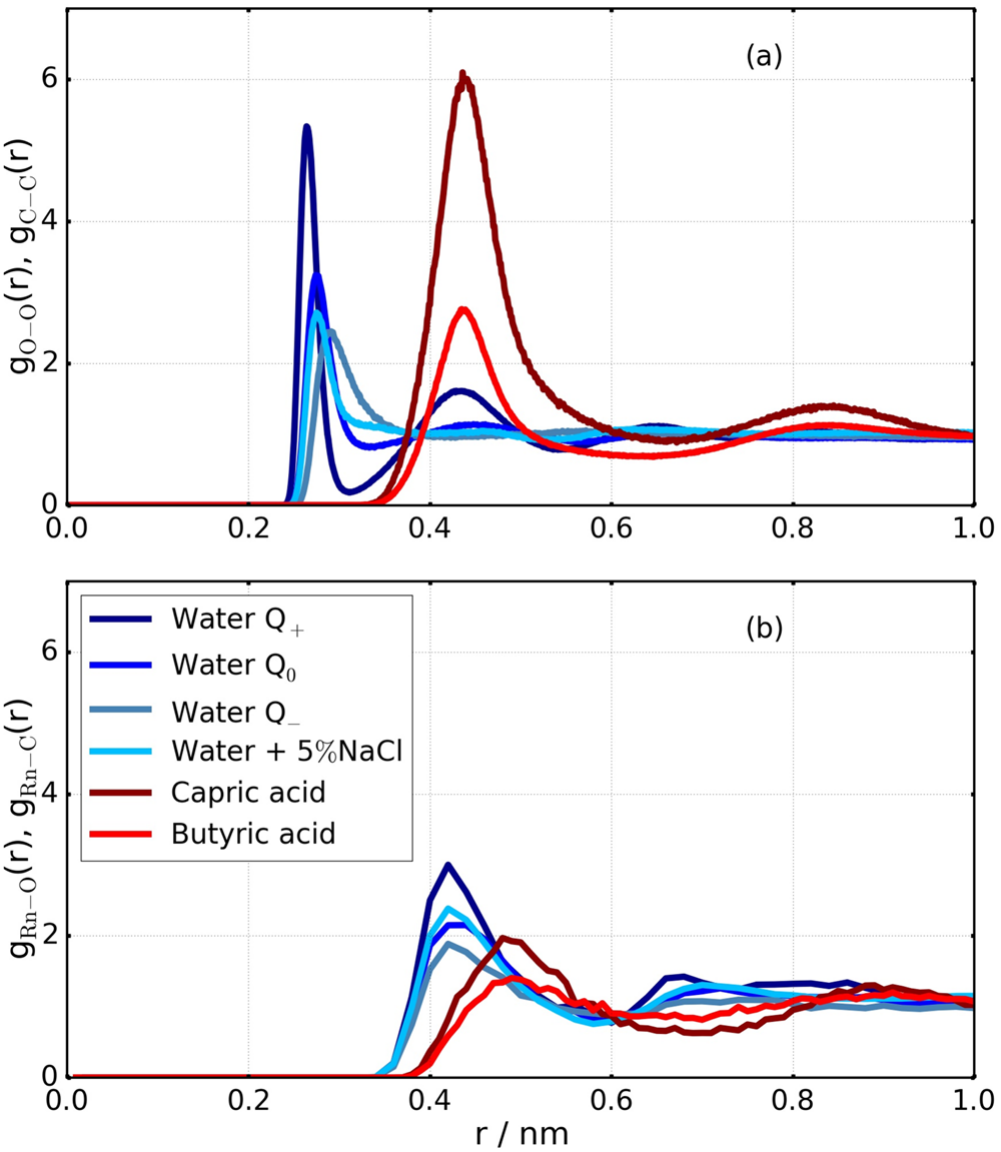
(**a**) Radial distribution function of the different types of water used in the simulations (based on the location of the oxygen atoms) and of fatty acids (based on the location of the first carbon atom). (**b**) Radial distribution function of the different solvent molecules around a solved radon atom, based again on the oxygen resp. carbon atoms.

In the following, we used the computer simulation data in order to explore how this concentration specific solubility difference emerges from the interaction of radon with the respective liquids. Since radon is a large atom, its presence interrupts the structure of the solvent molecules. Figure 1(a) shows the radial distribution functions (i.e., the distribution of intermolecular distances) of the different solvents in the absence of radon.

The first peak of each curve gives the nearest-neighbour distance in the non-distorted case. This distance is substantially smaller for water than for fat (which has larger molecules), and it decreases with increasing polarity, i.e., with increasing attractive interaction.

**Figure 2:**
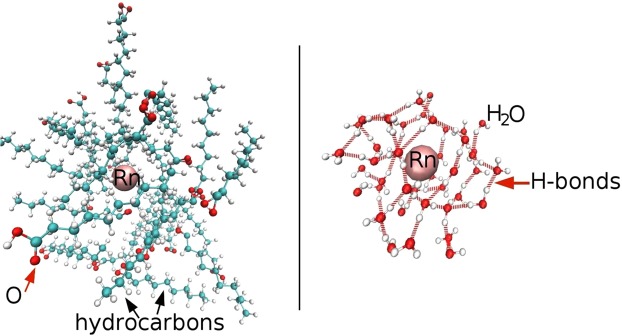
Snapshots showing the local structure of liquid molecules surrounding a radon atom, obtained when the system is equilibrated. Left: Capric acid molecules around a radon atom. Right: Water molecules surrounding a radon atom.

Figure 1(b) shows the radial distribution function of solvent molecules around radon atoms. The distance to the closest solvent atom is ≈0.4 nm, which is comparable to the radius of a radon atom. It is of the same order of magnitude as the distance between fatty acid molecules and considerably larger than the distance between water molecules. A visual impression of how the radon atom is embedded in the local structure is given in Fig. 2, which shows snapshots of the equilibrated system configurations obtained with MD simulations. The left panel shows capric acid molecules surrounding a radon atom, which is located in a void in the hydrocarbon arrangement. The right panel displays radon surrounded by water molecules. The surrounding water molecules are connected to each other by hydrogen bonds. In order to create space for the radon atom, the hydrogen bonds around it form a cage that has a different structure from the usual hydrogen bond network of pure water and thus this network is disturbed in the vicinity of the radon atom. Altogether, these observations indicate that the local structure of water is much more distorted by the presence of a radon atom than the structure of fatty acids. In order to penetrate water, the radon atom must modify the hydrogen bond network structure so that enough space for the radon atom is created, which goes along with an energy cost. Furthermore, in order to change from one location in water to a neighbouring one, the radon atom must break hydrogen bonds of its cage. A straightforward comparison between hydrogen bonds strength in water (between 21–24 kJ/mol)^19,20^ and the inter-molecular energy in hydrocarbons (related to the London dispersion energy on the order of 1–8 kJ/mol) suggests that the energy cost of breaking a bond is larger by one order of magnitude in water than in fat. Introducing radon in water thus comes with a high cost of free energy, leading to a low solubility. This low solubility is accompanied by a slow diffusion. Using our simulation data, we compared the diffusion of a radon atom in water with the diffusion of radon in hexane (see Table S1 of the Supplementary Information). The diffusion coefficient is at least four times lower in water compared to hexane. This supports our above finding that the diffusion of radon in water is hampered by a higher energy barrier due to the necessity of breaking hydrogen bonds. These two effects explain the much lower concentration specific solubility of radon in water compared to fat.

The amount of radon solved in water increases when the energy of the hydrogen bonds is reduced (Q_−_ water) or when the tetrahedral short range order of water is reduced by dissolving salt in water (see the concentration specific solubility data in Table 4(b)). A deeper theoretical understanding of the solvation of hydrophobic solutes is conveyed in the work of Sedlmeier *et al*.^21^.

In contrast to water, hydrocarbon aggregates provide free volume in which radon can enter without disrupting the molecular structure in a noticeable way. In the Supplementary Information (Fig. S1), we demonstrated this explicitly by showing the radial distribution of carbon in butyric and capric acid in two different cases: first in the liquid structure and second in the liquid after accumulation of radon. The effect of radon in the local structure of hydrocarbons is almost not visible, apart from a slight move of the shoulder of the first neighboring shell peak to larger distances. Interestingly, radon accumulates more in fatty acids than in linear oily chains. This may be related to the fact that fatty acids contain larger atoms (C and O), and that they have a heterogeneous structure with aliphatic groups that can form hydrogen bonds. In contrast, the difference between saturated and unsaturated fat molecules plays only a minor role for concentration specific solubility of radon (when taking into account the size of the error bars).

To conclude, our study explains the much better concentration specific solubility of radon in fat compared to water, and it has thus laid the ground for a more detailed exploration of the accumulation of radon in various tissues.

## Methods

### Experimental methods

The experiments were conducted in a radon chamber, and the samples were exposed at room temperature (295.2 ± 0.4 K) and atmospheric pressure (1001 ± 9 mbar) under controlled conditions (radon activity concentration, temperature and relative humidity)^22^. The most abundant fatty acids in the human body, oelic acid (C_18_H_34_O_2_), linolic acid (C_18_H_32_O_2_)^23^ and isotonic salt solution (0.9 mass percent NaCl) were exposed in the liquid phase. The radon activity concentration was constant during exposure.

**Figure 3:**
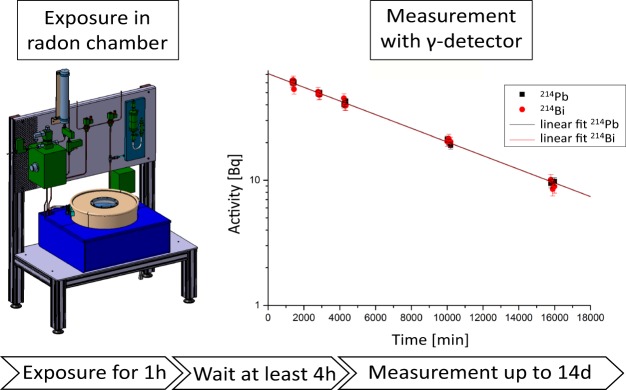
The samples were exposed for one hour in the radon chamber with ^222^Rn at room temperature and atmospheric pressure. Afterwards the samples were transferred into sealed glass jars and kept for four hours so that radioactive equilibrium between ^222^Rn and its daughter nuclei ^214^Pb and ^214^Bi could be reached. Subsequent measurement of the activities via *γ*-spectroscopy and by determining the activity at t = 0, the concentration specific solubility of radon could be determined by normalizing to the mass of the sample and the radon activity concentration during experiment.

The experimental scheme is shown in Fig. 3. Samples were placed in dishes with a layer thickness of around one centimetre and covered with a fibre glass filter for protection from contamination with radon decay products. Samples were exposed for one hour, in which saturation with ^222^Rn was reached, which was in agreement with diffusion measurements conducted in parallel^24^.

After exposure the radon chamber was flushed with air for five minutes to remove most of the radon. Then the specimen were transferred into uncontaminated glass jars and sealed. After approximately four hours a radioactive equilibrium between ^222^Rn and its daughter nuclei ^214^Pb and ^214^Bi was reached and their decay is governed by the lifetime of the primary radon. Their activity was determined by several *γ*-spectroscopic measurements of 15 minutes each within a period of up to 14 days.

The *γ*-spectra of the radioactive nucleides ^214^Pb and ^214^Bi were measured with a high purity Ge-detector. Data were analysed with commercial available software. After calibration for energy and efficiency, a background subtraction of an unexposed sample with the same geometry was performed. For efficiency calibration, the sample geometry was considered, as this has an impact on the self-absorption of the *γ*-quanta inside the specimen and on the solid angle of the emitted photons in relation to the detector. For analysis, the most prominent *γ*-lines at 242 keV, 295 keV, 352 keV (all ^214^Pb) and 609 keV (^214^Bi) were taken into account. Subsequently the results were plotted over the time after exposure. The intersection of the extrapolated activity with the y-axis gives the initial activity of the measured isotopes and therefore the initial ^222^Rn concentration. By normalizing to the mass of the sample and considering the density of the sample material, the activity inside the specimen was determined. Taking into account the radon activity concentration during the experiment, the concentration specific solubility of ^222^Rn in the sample was calculated.

### Computational methods

Classical MD simulations are performed with the NAMD^25^ 2.10 simulation package. The SPC/E (extended simple point charge)^26^ model is used to model liquid water, where a water molecule is represented by three atom sites. The partial charge attached to the oxygen atom is *Q*_0_ = −0.8476*e* and half i.e 0.423*e* is attached to the hydrogen atoms. In order to vary the polarity, the partial charges attached on the oxygen atom are varied and values *Q*_0_ = −0.8476*e*, *Q*_±_ = *Q*_0_ ± 15%*Q*_0_ are used.

Alkaline solutions of 5% of NaCl and 1.9% of CaCl_2_ are used in order to imitate the ambient liquid in human body cells. The salt concentrations are defined with respect to molar percentage but the mass percentage are comparable since CaCl_2_ is roughly two times heavier than NaCl. The salt concentrations here defined are higher than the physiological density (0.9%) but are the ones generally used in MD simulations to characterize dynamical properties of ions in salt solutions^27^. Interaction parameters of the alkaline ions were extracted from the work of Luo and Aqvist^28,29^.

Additionally, fat is mimicked by simulating the linear isomer of hexane (C_6_H_14_), butyric (C_4_H_8_O_2_) and capric (C_10_H_20_O_2_) acid. The parameters used to simulate hexane are taken from the CHARMM22 force field^30^, and those for butyric and capric acid from the work of Clifford *et al*.^31^. Radon atoms interact with the atoms of each liquid only via Van der Waals interaction, implemented as Lennard-Jones (LJ) potential

1 $${U}_{{\rm{LJ}}}=4\epsilon (\frac{{\sigma }^{12}}{{r}_{i,j}^{12}}-\frac{{\sigma }^{6}}{{r}_{i,j}^{6}}).$$

The isotope ^222^Rn is used to model radon. In order to obtain the LJ parameters corresponding to the gaseous phase of radon, the well depth $\epsilon$ is derived from the radon bulk cohesive energy^32^ $({{\rm{U}}}_{{\rm{coh}}}):\frac{{{\rm{U}}}_{{\rm{coh}}}}{{N}_{0}}=-\,(2.15)\cdot 4\epsilon$ for ^222^Rn. The value of *σ* used in the present study has been chosen accordingly in order to reproduce the correct density of radon gas. Some simulation using the parameters suggested by other authors^33–36^ have been performed, but the resulting density at room temperature was at least 5% higher than the empirical radon density.

The interaction of radon with other atoms occurs also via the LJ potential, with parameters calculated using the Lorentz-Berthelot mixing rules^37^.

Table 5 shows all LJ parameters as well as partial charges assigned to the different atom types. All runs have been carried out within the NPT ensemble keeping the pressure constant and equal to 1 bar using the Langevin-Piston method^38^. Whereas the temperature is fixed to room temperature (T = 298 K) using a Langevin thermostat^25^ with a coupling coefficient of 1.0 ps^−1^. An integration time step of 1 fs is utilized in order to accurately follow the motion of fast radon atoms. The simulations have been run for approximately 10 ns for the equilibration of each pure liquid, meanwhile the accumulation of radon in each liquid has been observed during a time interval of at least 2 ns. We used periodic boundary conditions, allowing the calculation of the long-range Coulomb electrostatic interactions with the particle-mesh Ewald summation, using a cut-off of 1.5 nm and a switching distance of 1.2 nm.

**Table 5:** LJ parameters for each atomic site used in the simulation.

Atoms	${\boldsymbol{\epsilon }}$ (Kcal/mol)	*σ* (nm)	q_*i*_ (e)
O_W_	0.155	0.317	−0.847
H_W_	0.000	0.179	0.423
C_*e*_	0.078	0.363	−0.270
C_*b*_	0.0560	0.358	−0.180
H_*e*_	0.024	0.238	0.009
H_*b*_	0.035	0.238	0.009
C	0.081	0.390	0.750
O=(C)	0.156	0.305	−0.550
O–(H)	0.184	0.302	−0.610
H	0.000	0.179	0.009
CH_2_	0.091	0.395	−0.180
CH_3_	0.194	0.375	−0.270
N_a_	0.046	0.251	1.000
Cl	0.150	0.404	−1.000
C_a_	0.023	0.324	2.000
Rn	0.541	0.453	0.000

O_W_ and H_W_ stand for the oxygen and hydrogen atoms of water. C_*b*_ and C_*e*_ are the carbons of the hexane chain linked respectively to 2 (H_*b*_) and 3 (H_*e*_) hydrogens. C, O=(C) and O–(H) are the atoms of the fatty acids carboxylic group. CH_2_ and CH_3_ are the carbons of the fatty acids linked respectively to 2 and 3 hydrogen atoms, and H represent the hydrogens. Na, Ca and Cl represent the sodium, calcium and chloride ions.

The number of molecules used in the simulations are 9999 for water, 3479 for hexane, 3375 and 1122 for butyric and capric acid respectively, and 9702 for radon. The first set of simulations have been done for pure systems only in order to check how good are the interaction parameters in reproducing the expected density at normal pressure and room temperature. The second set of simulations, which have been used for measuring concentration specific solubility of radon, started with a liquid droplet surrounded by 159 radon atoms.

## Supplementary information

A combined experimental and theoretical study of radon solubility in fat and water
