# Supplementary material for: A combined experimental and theoretical study of radon solubility in fat and water
Source: Sci Rep. 2019 Jul 24;9:10768. doi: 10.1038/s41598-019-47236-y (PMC6656752; doi:10.1038/s41598-019-47236-y)
Supplement: Supplementary file 1 — A combined experimental and theoretical study of radon solubility in fat and water [file 41598_2019_47236_MOESM1_ESM.pdf]

A combined experimental and theoretical study of radon solubility in fat and water

Elvira P. Sanjon[1,+], Andreas Maier[2,*,+], Annika Hinrichs[2,3], Gerhard Kraft[2], Barbara Drossel[1,+], and Claudia Fournier[2,+]

[1]Institut für Festkörperphysik, Technische Universität Darmstadt, Hochschulstr. 6, 64289 Darmstadt, Germany
[2]GSI Helmholtzzentrum für Schwerionenforschung, Biophysics division, Planckstr. 1, 64291 Darmstadt, Germany
[3]Goethe Universität Frankfurt, Max-von-Laue-Str. 1, 60438 Frankfurt, Germany
[*]a.maier@gsi.de
[+]these authors contributed equally to this work

In this supplementary information, we provide calculated diffusion coefficients of radon in in different liquids. Additionally, we added the local structure of the first carbon atom in fatty acids before and after radon accumulation.

Systems	Diffusion
	$(m^2 \cdot s)$
Water Q_+	$1.3 \cdot 10^{-9}$
Water Q_0	$1.7 \cdot 10^{-9}$
Water Q_-	$5 \cdot 10^{-9}$
C_6H_{14}	$6.7 \cdot 10^{-9}$
Rn	$1.10 \cdot 10^{-5}$

Table S 1. Diffusion coefficients of one radon atom in the different liquids or in bulk radon. The simulations have been performed at normal pressure p = 1 bar and room temperature T = 298 K.

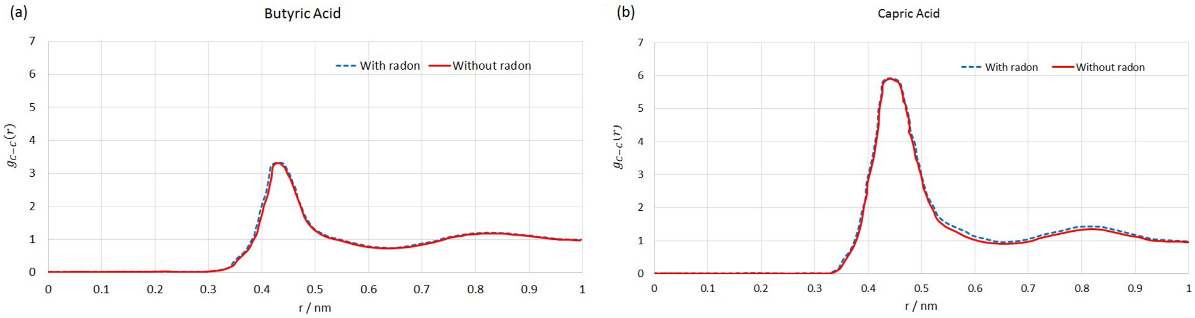

Figure S 1. (a) Radial distribution function of the first carbon atom of butyric acid (a) and capric acid (b). The solid lines correspond to the case in which the liquid is simulated without radon and the dotted lines correspond to the liquid after radon accumulation.
